# [F-18] FDG-PET/CT parameters as predictors of outcome in inoperable NSCLC patients

**DOI:** 10.1515/raon-2015-0043

**Published:** 2015-11-27

**Authors:** Antonio Nappi, Rosj Gallicchio, Vittorio Simeon, Anna Nardelli, Alessandra Pelagalli, Angela Zupa, Giulia Vita, Angela Venetucci, Michele Di Cosola, Francesco Barbato, Giovanni Storto

**Affiliations:** 1 Nuclear Medicine Department, Istituto di Ricovero e Cura a Carattere Scientifico (IRCCS), Centro di Riferimento Oncologico di Basilicata (CROB), Rionero in Vulture, Italy; 2 Research Laboratories, Istituto di Ricovero e Cura a Carattere Scientifico (IRCCS), Centro di Riferimento Oncologico di Basilicata (CROB), Rionero in Vulture, Italy; 3 Istituto di Biostrutture e Bioimmagini, Consiglio Nazionale delle Ricerche, Napoli, Italy; 4 Università degli Studi di Foggia, Italy; 5 CMO Oplonti Medical Centre, Italy

**Keywords:** Non-small-cell lung cancer, [F-18] FDG PET/CT, quantitative assessment, glycolytic activity, survival

## Abstract

**Background:**

We evaluated the prognostic significance of standardized uptake value (SUVmax), metabolic tumour volume (MTV), and total lesion glycolysis (TLG) in [F-18] FDG PET/CT findings in patients with inoperable non-small-cell lung cancer (NSCLC).

**Patients and methods.:**

One hundred and three patients (mean age, 65.6 ± 16 years) underwent [F-18] FDG PET/CT before the chemotherapy. The SUVmax value, the MTV (cm^3^; 42% threshold) and the TLG (g) were registered. The patients were followed up to 18 months thereafter (range 12–55 months). Failure to respond without progression, progression and/or disease-related death constituted surrogate end-points. The optimal SUVmax, MTV and TLG cut-off to predict the patients’ outcome were estimated. PET/CT results were then related to disease outcome (progression free survival; PFS).

**Results:**

The Kaplan-Meier survival analysis for SUVmax showed a significant shorter PFS in patients presenting with lower values as compared to those with higher (p < 0.05, log-rank test). MTV and TLG were not suitable for predicting PFS apart from the subset of patients with mediastinal nodal involvement.

**Conclusions:**

Despite the availability of new tools for the quantitative assessment of disease activity on PET/CT, the SUVmax rather than MTV and TLG remains the only predictor for PFS in NSCLC patients. MTV holds a value only when concomitant nodal involvement occurs.

## Introduction

Lung cancer constitutes the most common cause of cancer death around the world and is the second most common gender unrelated cancer.^1^–^4^ Non–small-cell lung cancer (NSCLC) includes up to 85% of all lung cancer cases.^1^ The treatment and prognosis of NSCLC depend mostly upon the stage outlined according to the American Joint Committee on Cancer (AJCC) staging system.^1^,^5^–^7^ Although NSCLC remains a deadly cancer, to identify prognostic factors represents a clinical challenge since a modulated therapy is still possible. The TNM staging as well as the stage grouping (I–IV) have been largely disputed so far. Other patients’ specific factors such as age, pulmonary performance, and co-morbidity might also influence the selection for treatment options.^1^,^5^ In fact, for the early-stage NSCLC, surgical resection is the standard of care, whereas in patients with unresectable, locally advanced tumour, as per stage III NSCLC, the chemotherapy in combination with radiation therapy represents the best option.^7^–^9^ Systemic chemotherapy is reserved for the stage IV patients.^10^–^12^ Over the paste decade FDG (18F-fluoro-2-deoxy-D-glucose) PET/CT has demonstrated to be a powerful tool for staging and assessment of the treatment response in patients suffering from NSCLC.^13^,^14^ Even if PET/CT is widely used in this setting, only limited data are presently available to describe in concert the role of PET/CT quantitative parameters for the prediction of the disease outcome (with special emphasis to those recently introduced).^15^,^16^ The findings are discordant being alternatively allotted as valuable or worthless.^17^–^19^ At this time, the metabolic tumour volume (MTV) and the total lesion glycolysis (TLG) are measures of the metabolic activity of tumours derived from the [F-18] FDG up-take on PET/CT images. Initial data have recently addressed their value in NSCLC.^20^ The MTV and the TLG can be easily calculated in the primary tumour by means of a segmentation technique. The manual or semiautomatic measurement of the pre-treatment MTV has been shown to be better than SUVmax for predicting patients prognosis in different solid neoplasms such as head and neck cancer, with or without metastases.^21^,^22^ Our study was undertaken to investigate the relationship between the functional tumour parameters at staging (SUVmax, MTV, and TLG) and the progression free survival in inoperable patients with NSCLC, presenting with stage IIIB/IV, for whom a chemo-therapy (alone or in combination with radiotherapy) was shortly planned.

## Patients and methods

### Patients

Two hundred and ninety patients suffering from inoperable NSCLC were referred for a staging PET/CT scan prior to the start of chemotherapy between January 2008 and January 2012. Among them 103 (17 women; mean age 65,6 ± 16 years) strictly fulfilled inclusion criteria, which were: age at entry of 18 years or older; negative pregnancy test; stage IIIB and stage IV without distant metastases out of chest (inoperable tumour, CT staged, histologically proven). Patients whose lesions were excised surgically before PET/CT imaging or who had received neo-adjuvant chemo-radiotherapy within the preceding 6 months prior to PET scan were excluded from this study.

Individual data are summarized in Table 1. Our institutional review board provided approval for the procedures included in the study. All patients who underwent PET/CT scan signed an informed consent form in accordance with the Declaration of Helsinki.

### Imaging technique

The patients were well-hydrated before receiving [F-18] FDG intravenously (444–555 MBq). Sixty minutes after the tracer injection, PET and CT scans were obtained using a commercial PET/CT scanner (GE Discovery VCT scanner; Waukesha, WI) that combines a PET scanner and a Light Speed VCT sixty-four row MDCT system. MDCT (pitch_x_ 1.5; 120 mAs; 120 kVp) was performed without the use of an intravenous and/or oral contrast medium. The PET scanning was subsequently performed, acquiring 4 minutes per bed position and six to eight bed positions per patient, depending on patient height. The raw CT data were reconstructed into transverse images with a 3.75-mm section thickness. Sagittal and coronal CT images were generated by reconstruction of the transverse data. Raw PET data were reconstructed with and without attenuation correction into transverse, sagittal, and coronal images. Attenuation correction was based on the CT attenuation coefficients, which were determined by iterative reconstruction. The patients were kept fasting for at least 6 hours prior to the imaging. Blood glucose levels were measured in all patients before [F-18] FDG administration; patients with values above 7.77 mMol/L (140 ml/dl) were excluded from examination.

### Imaging evaluation

All images were reviewed by using a PET/CT fusion software (Volumetrix for PET-CT, GE Healthcare, Waukesha, WI, USA). Each PET/CT study was interpreted by two experienced nuclear medicine physicians (G.S., and A.N., each with fifteen years of expertise); one of them also being a board certified radiologist. They were blinded to the patient histories. The examiners first evaluated the CT images alone. The primary lesion size was visually estimated and measured for minimum and maximum diameters by using vendor-provided software. A primary lesion was defined as solitary pulmonary nodule or peripheral nodule with an ill-defined, irregular, and spiculated border as well as identifiable mass or densitometric modifications of lung parenchyma (as per tissue thickening with or without air bronchogram findings and pseudocavitation/focal lucency, ground-glass opacification). Peribronchial/mediastinal lymph nodes larger than 1 cm in minimum diameter and with altered shape or hilus, assessed with chest window CT settings, were considered suspicious. The PET studies were evaluated both visually and semi-quantitatively. Then, the maximum standardized uptake values and body weight corrected (SUVmax) as well as the metabolic tumour volume (MTV; cm^3^; 42% threshold) and total lesion glycolysis (TLG; g) were determined by using the same vendor-provided software for the primary lesion. The MTV was defined as the volume with SUV over 42% of SUVmax. TLG was calculated as the product of SUVmean and MTV (TLG = SUVmean × MTV). Finally, lymph nodes were evaluated systematically according to the topographic criteria on the PET/CT scan using the SUVmax to determine their metabolic activity, if any.

As previously reported, a conventional SUVmax cut-off value of 2.5 (settled in studies using ROC curve analysis) has been considered to provide excellent specificity and sensitivity for detecting lesions.^15^,^19^,^23^ Accordingly, a significant uptake was reported to be higher than 2.5.

### Follow-up assessment

The patients were categorized into two groups according to SUVmax, MTV and TLG cut-off points determined by receiver-operator-curve (ROC) analysis. The performance status and the status of disease were followed up to 18 months thereafter (range 12–55). The evaluation was carried out by means of clinical and laboratory parameters during scheduled or unscheduled visits, on the basis of diagnostic imaging (*i.e*. CT) results, if any, as well as by phone interview. Failure to respond without disease progression, disease progression after four cycles of chemotherapy and/or disease-related death were defined as surrogate end-points. Disease progression was identified as documentation of a new lesion or enlargement of a previous existing lesion; when there was missing information, the date of unscheduled new, alternative treatment was considered. Clinical parameters and PET/CT results were then correlated to the progression free survival (PFS). PFS was defined as the time from PET/CT until end point occurrence or the time of last censor.

### Statistical analysis

Continuous data were expressed as the mean ± 1 SD and median, as appropriate. Comparisons between the mean values were performed with an unpaired Student’s t test (two-tailed probability) or Wilcoxon rank-sum test, according to skewness and kurtosis test for normality test. The ROC analysis was performed to estimate the optimal cut-off of SUVmax, MTV and TLG for differentiating patients at high risk of end-points occur-rence. Sensitivity and specificity were computed according to the standard method. Both univariate and multivariate regression analysis were used. A multivariate binary logistic regression analysis (enter method) was used to test the chosen (homogeneously available) independent variables such as age, gender, the presence of loco-regional nodal involvement and the lesion SUVmax, MTV and TLG for their association with unsuccessful outcome. The PET/CT results were then correlated to the PFS. The Kaplan-Meier method was used to plot PFS. The predefined cut-off point for SUVmax, MTV and TLG were adopted, and the curves were compared by log-rank testing. Survival analysis was performed by univariate Cox proportional hazard regression analysis. A probability (p) value < 0.05 was considered statistically significant.

## Results

### Patient characteristics

Patients with NSCLC were evaluated during the study period. Among those enrolled, none had a surgical intervention. All the patients were wild-type (no EGFR mutations, no ALK rearrangements detected) and underwent chemotherapy (from 4 to 6 cycles) after the PET/CT study; they were treated with cisplatin-based regimens with or without radiation therapy according to established dose or fractionation schedules up to 60 Gy–70 Gy (in 6–7 weeks). Overall individual patient characteristics are reported in Table 1.

### Energetic turnover measurements

The PET/CT was reported as positive in all patients (i.e. significant uptake; visually detectable and SUV higher than 2.5). The median SUVmax value was 7.3 (range 2.7–44), median MTV was 16.5 cm^3^ (range 3.7–38.1) and median TLG was 274.5 g (range 72.6–1039.9). According to nodal status, the median SUVmax value was 4.6 (range 2.7–23) and 10.7 (range 2.5–44) in patients without and with nodal involvement, respectively (p < 0.05), whereas median MTV was 15.2 (3.7–88) cm^3^ and 18.6 (5.1–38.1) cm^3^, respectively (p = 0.07). TLG values were not significantly different as well.

### Prediction and discriminating values of SUVmax, MTV and TLG

**FIGURE 1.**
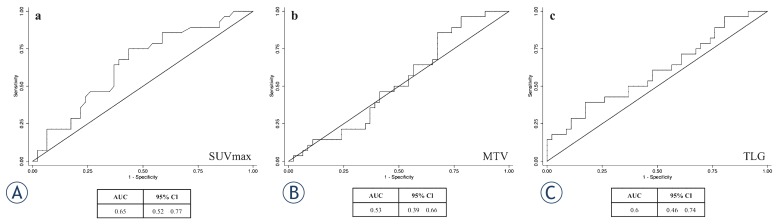
ROC curve analysis establishing the cut off value of SUVmax, MTV and TLG for predicting Progression Free Survival. The cut off value of SUVmax **(A)**, MTV **(B)** and TLG **(C)** for stratifying patients was 6.3, 8.4 (cm^3^) and 259 (g), respectively.

The ROC curve analysis recognizing the cut off value of SUVmax, MTV and TLG is showed in Figure 1. The area under the curve (AUC) for SUVmax was 0.64 and 6.3 was established as the cut off value. The AUC for MTV and TLG was 0.52 and 0.6, respectively whereas MTV and TLG cut off values were 8.4 and 259.0, respectively. The sensitivity of SUVmax cut-off on PET/CT for predicting the outcome was 75% whereas specificity was 57%. The sensitivity and specificity of MTV and TLG cut-off were 85% and 33%, and 61% and 52%, respectively. At univariate logistic regression analysis both the SUVmax (p < 0.01; OR 3.9) and presence of nodal involvement (p < 0.001; OR 4.3) were predictive of the end-points, whereas MTV, TLG, age and gender were not. At multivariate logistic regression analysis only the presence of loco-regional nodal involvement on PET/CT contributed to the prediction of the end points occurrence (p < 0.05; OR 3.2), whereas MTV, TLG, age and gender did not. The SUVmax showed a trend for predicting the outcome (p= 0.08; OR 2.6), (Table 2).

### Clinical end points and follow up

Overall 28 of 103 patients (27%) reached the endpoint, 12 experienced no response/progression (43%) and 16 (57%) died. The mean SUVmax value in patients who reached an end point was higher as compared to those who did not (11.2±4.4 vs 8.4±5.9; p < 0.05, Wilcoxon rank-sum test).

**FIGURE 2.**
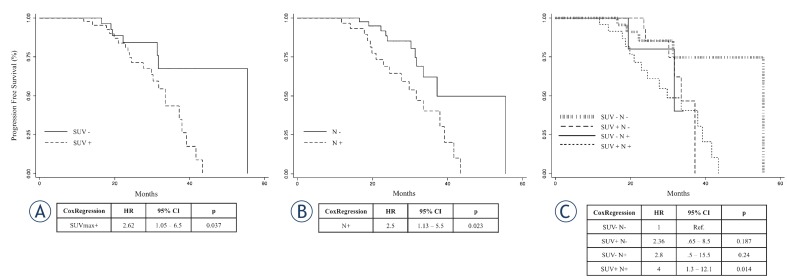
Kaplan-Meier survival graphs indicate a significant difference in PFS between the group of patients categorized by SUVmax. **(A)** Kaplan-Meier graph of SUVmax and PFS showing SUVmax above (solid line) and below (dotted line) the cut off of 6.3. **(B)** Kaplan-Meier graph of lymph-nodal involvement and PFS without (N-, solid line) and with (N+, dotted line) nodal concern. **(C)** Survival by combination of SUVmax and nodal positivity. Kaplan-Meier graph of both SUVmax and nodal positivity and Progression Free Survival. SUVmax+ and SUVmax- indicate values of SUVmax above and below the cut off value of 6.3, respectively. N+ and N- indicate the presence and the absence of locoregional lymph-nodal involvement discovered at PET/CT.

The median follow up was 18 months (range 12–55 months). The Kaplan-Meier survival analysis for SUVmax showed a significant difference in PFS (p < 0.05, log-rank te st). Shorter PFS was observed in patients with lesion SUVmax over 6.3 as compared to those with SUVmax values below this value (median survival: 33 vs 41 months; p < 0.05, cox regression) (Figure 2a). Patients with nodal involvement showed a shorter PFS as compared to those who did not (median survival: 30 vs 37 months; p < 0.05, cox regression) (Figure 2b). When nodal involvement was aggregated to higher SUVmax the patients showed a shorter PFS as compared to those without nodal involvement and lower SUVmax (Figure 2c).

Noteworthy, in the sub-group with nodal involvement, patients with lower MTV showed better outcome as compared to those with higher MTV value (median MTV 10.8; range 5.1–40 vs 17.6; range 9.7–381; p < 0.05). At ROC curve analysis for this subgroup the AUC was 0.74 and 10.9 was the cut off value. The sensitivity and specificity of MTV cut-off on PET/CT for predicting the outcome were 94% and 54%, respectively. However, in this subgroup the MTV constituted a risk factor when settled as continuous variable (hazard ratio 1.03 per unit increment; p < 0.05; 95% CI: 1.005–1.060), but was not able to predict PFS once used as dichotomous variable (hazard ratio 2.14; p = 0.47; 95% CI: 0.27–17.3).

## Discussion

Current treatment approach based on stage of the disease (operable and inoperable tumours) is not entirely satisfactory in NSCLC, whilst the implementation of more novel therapeutic strategies targeting specific receptors continues to hold great promise.^24^ This distressed scenario imposes the re-weighting of the prognostic factors available, especially those allowing the correct understanding of tumour biology and its therapeutic sensitivity.^25^ Despite the efficacy of the new therapeutic agents, the necessity arises for directing their strength in accordance to the tumour intrinsic features since metabolic pathways differ from subject to subject, claiming for a further specific treatment approach. Nowadays, [F-18] FDG PET/CT provides a global assessment of NSCLC patients for staging, re-staging and therapy evaluation, having a prognostic value as well.^26^ In addition, the PET/CT methodology allows a reproducible semi-quantitative assessment by means of several uptake indices, including the estimate of the whole tumour burden.^20^ Among the PET parameters, SUVmax is the most commonly used. It reflects tumour glucose metabolism of the most aggressive cell component, given that previous studies have suggested the association between SUVmax and tumour aggressiveness.^27^ Conversely, the metabolic tumour volume as well as the total lesion glycolysis allow to estimate the tumour energetic turn-over throughout the volume of the lesion above a minimum threshold designed to exclude background activity.

Since all the quantitative parameters derived from PET/CT have not yet been comprehensively investigated as prognostic factors in inoperable NSCLC patients, we evalua ted, in a similar setting, the predictive impact of SUVmax, MTV, TLG in NSCLC patients, before radio-chemotherapy.

The main finding of the present study is that SUVmax constitutes the only metabolic parameter, among the others, able to predict the progression free survival in inoperable NSCLC patients having stage IIIB or chest confined stage IV, whereas MTV holds a slightly but predictive value only in case of loco-regional lympho-nodal involvement.

According to previous studies, patients presenting with higher values of SUVmax showed a poor outcome as compared to those having lower, indicating that the high magnitude of glycolytic activity, rather than the extent of metabolic tumour burden, predicts a poor response to subsequent therapy.^28^ As a result, lower SUVmax values showed a virtually “protective” significance, whereas the MTV and the TLG did not impact on the outcome of patients. Unfortunately, this dichotomous standpoint does not stratify the grey zone of patients discovered/confirmed to have nodal metabolic load (at PET/CT finding). Such an aspect has not be en assessed, if not partially, in previous studies.^29^ The patients who showed nodal involvement associated to higher SUVmax had similar PFS to those presenting lower SUVmax values. As expected, the “protective value” of a low SUVmax fails when a lymph nodal involvement occurs; the quote of our patients with nodal involvement had a poor outcome irrespective of SUVmax values. It is well known, however, that, apart from distant metastases, positive mediastinal lymph nodes have the most significant prognostic value for recurrence and death in NSCLC patients. Interestingly, the MTV acquired a prognostic significance in this sub-setting since it was slightly, but significantly associated with the outcome. This finding could be related to the impact of the amount of metabolically active burden when multiple lesions are concomitantly present, which better explains the risk of mediastinal nodal metastases.^30^ Some authors have already reported similar results but in more compromised, surgically treated patients whose nodal involvement was confirmed by histology and, not by PET/CT.^29^ Additionally, they described the incremental risk of developing nodal involvement due to MTV whereas in our setting it was related to PFS.

From a patho-physiological point of view, this finding is not surprising since SUVmax represents an index of cell glucose utilization and substantially reflects the first step of aerobic glycolysis, including cell uptake, whilst MTV better depicts the comprehensive metabolic tumour burden.

Considering the reasonable natural history of the NSCLC, it could be envisaged that the persistent metabolism of glucose to lactate even in aerobic conditions is an adaptation to intermittent hypoxia in initial lesions, those supposed to be not yet metastatic (see SUVmax significance). However, once upregulation of glycolysis has taken place, cell populations acquire acid resistant phenotypes and powerful growth advantage. Such a condition is presumably thought to favour further, continuous hypoxia moving tumour and non-tumour cells automatically toward a type of anaerobic respiration. As a consequence, SUVmax depicting the sole hottest pixel within a tumour, might no longer indicate the whole tumour burden. On the other hand, the MTV, which embodies a volumetric representation of the metabolic charge, out of the more active pixel, seems to acquire a prognostic significance when tumour has dimensionally progressed and metastasized giving an incremental risk of event per unit increase.

From a methodological point of view, it is conceivable that the lack of actually standardized criteria for determining MTV and TLG implies that these volumetric measurements might not replicate the absolute values produced elsewhere and continue to hold some intrinsic limits. However, several studies are being performed accounting for and validating their value.

The prognostic impact of F-18 FDG PET/CT quantification parameters in non-surgical NSCLC patients remains to be completely established. In view of that, our results demonstrate, as a rule, that SUVmax is a better predictive indicator of progression free survival than new volumetric parameters even if the MTV purchases a prognostic power in case of concomitant nodal metabolic concern. These findings endorse the idea that metabolic quantification parameters may be implemented in daily practice redirecting the concept that size (morphologically assessed) and histology constitute the only key-points for determining the biological aggressiveness of lung cancer.

This study included NSCLC patients that strictly fulfilled inclusion criteria (non-operable tumour) and it was performed in a single centre, which gives reason for the relatively small samples size.

## Conclusions

The quantitative assessment by F18-FDG PET/CT quantification parameters may be helpful to manage inoperable NSCLC patients before chemotherapy. In this setting, the magnitude of glycolytic activity rather than the tumour burden extent seems to be generally predictive of response to subsequent therapy apart from a loco-regional metastatic concern.

## Figures and Tables

**TABLE 1.** Overall patient characteristics

**Number**	**103**
Age at diagnosis, years, median (range)	68 (48–79)
Gender ratio (M : F)	86 :17
Histological type	
Adenocarcinoma (%)	67 (65)
Squamous cell carcinoma (%)	27 (26)
Broncho-alveolar carcinoma (%)	5 (5)
Adeno-squamous carcinoma (%)	2 (2)
Others (%)	2 (2)
Stage at diagnosis	
IIIB (%)	75 (73)
IV (%)	28 (27)
Chemotherapy (%)	22 (21)
Radiotherapy (%)	21 (20)
Radio-chemotherapy combination (%)	60 (59)
Locoregional/mediastinal lymph node involvement	
Yes (%)	63 (61)
No (%)	40 (39)
Final patient status	
No response/Progression (%)	12 (43)
Death (%)	16 (57)

**TABLE 2.** Logistic regression analysis

	**Risk of progression**					
	
	**Univariate analysis**			**Multivariate analysis**		

	**OR**	**95% CI**	***p***	**OR**	**95% CI**	***p***
**Age**	0.95	0.9–1.007	0.093	-	-	-
**Gender**	2.25	0.75–6.76	0.149	-	-	-
SUVmax^*^	3.9	1.38–11.0	**0.010**	2.65	0.87–8.06	0.085
MTV^*^	2.9	0.85–9.88	0.088	-	-	-
TLG^*^	2.6	0.89–7.81	0.080	-	-	-
**Lymph-node**	4.37	1.6–11.95	**0.004**	3.2	1.09–9.25	**0.034**

* Dichotomized variables on ROC analysis basis;

OR = odds ratio; CI = confidence interval; SUVmax = standardized uptake value; MTV = metabolic tumour volume; TLG = total lesion glycolysis.
